# Looking for Celiac Disease in Italian Women with Endometriosis: A Case Control Study

**DOI:** 10.1155/2014/236821

**Published:** 2014-03-31

**Authors:** Luca Santoro, Sebastiano Campo, Ferruccio D'Onofrio, Antonella Gallo, Marcello Covino, Vincenzo Campo, Guglielmo Palombini, Angelo Santoliquido, Giovanni Gasbarrini, Massimo Montalto

**Affiliations:** ^1^Department of Internal Medicine, Catholic University of Rome, 00168 Rome, Italy; ^2^Integrated Complex Columbus-Gemelli Hospital, Department of Internal Medicine, Catholic University of Rome, Via Giuseppe Moscati 31, 00168 Rome, Italy; ^3^Department of Obstetrics and Gynaecology, Catholic University of Rome, 00168 Rome, Italy; ^4^Institute of General Pathology, Catholic University of Rome, 00168 Rome, Italy

## Abstract

In the last years, a potential link between endometriosis and celiac disease has been hypothesized since these disorders share some similarities, specifically concerning a potential role of oxidative stress, inflammation, and immunological dysfunctions. We investigated the prevalence of celiac disease among Italian women with endometriosis with respect to general population. Consecutive women with a laparoscopic and histological confirmed diagnosis of endometriosis were enrolled; female nurses of our institution, without a known history of endometriosis, were enrolled as controls. IgA endomysial and tissue transglutaminase antibodies measurement and serum total IgA dosage were performed in both groups. An upper digestive endoscopy with an intestinal biopsy was performed in case of antibodies positivity. Presence of infertility, miscarriage, coexistence of other autoimmune diseases, and family history of autoimmune diseases was also investigated in all subjects. Celiac disease was diagnosed in 5 of 223 women with endometriosis and in 2 of 246 controls (2.2% versus 0.8%; *P* = 0.265). Patients with endometriosis showed a largely higher rate of infertility compared to control group (27.4% versus 2.4%; *P* < 0.001). Our results confirm that also in Italian population an increased prevalence of celiac disease among patients with endometriosis is found, although this trend does not reach the statistical significance.

## 1. Introduction

Endometriosis is a chronic gynaecologic disorder characterized by the presence of endometrial tissue outside the uterus, mainly in the pelvic cavity. It is estimated to affect at least 5–10% of women in the reproductive age, up to 40–80% of women complaining of pelvic pain, and up to 30–50% of infertile patients [1, 2]. The pathogenesis of endometriosis is still under active investigation, and in the last few years a growing amount of data has underlined the potential role of oxidative stress, inflammation, and immunological dysfunctions in its development [3, 4]. It is noteworthy that these features seem to be not restricted only to peritoneum, being found also in the peripheral blood, so that endometriosis can be considered, in effect, as a systemic disease with a widespread inflammatory status that could also explain extrapelvic locations of endometriosis and its association with other diseases [5–9].

Celiac disease (CD) is an autoimmune enteropathy, occurring in genetically susceptible individuals, induced by the ingestion of gluten-containing foods and characterized by intestinal malabsorption and total or subtotal atrophy of intestinal villi [10]. Recent reports indicated that CD prevalence is growing, up to 2% in some Western countries [11]; in Italy a prevalence of 0.5–1% is referred to in general population [12, 13]. It is well known that CD can be associated with other intestinal and extraintestinal diseases, in particular with autoimmune disorders, such as type 1 diabetes mellitus, autoimmune thyroiditis, rheumatoid arthritis, and Sjögren's syndrome [14–16].

Very recently, some first studies have hypothesized a potential link between endometriosis and CD, since these conditions share some similarities [17, 18]. In our study, we investigated for the first time the prevalence of CD among Italian women with endometriosis with respect to general population.

## 2. Materials and Methods

Consecutive women with a laparoscopic and histological confirmed diagnosis of endometriosis referring to the Department of Obstetrics and Gynaecology of Catholic University of Rome between January 1, 2012, and December 31, 2012, were considered for the study. Endometriosis severity was classified according to the American Society for Reproductive Medicine revised classification of endometriosis [19]. Female nurses of our institution, without a known history of endometriosis, were enrolled in the study as control group.

At enrollment, a venous blood sample was collected by either patients or controls for IgA endomysial (EMA) and tissue transglutaminase (t-TGA) antibodies measurement and serum total IgA dosage. Qualitative and semiquantitative detection of EMA was performed using commercially available indirect immunofluorescence antibody test (ImmuGlo, IMMCO Diagnostics, Buffalo, NY); the presence of a characteristic pattern to fluorescence microscope was scored as positive for CD. For the detection and quantification of t-TGA antibodies, commercially available ELISA kit was used (IMMCO Diagnostics, Buffalo, NY); a t-TGA titre of >25 U/mL was considered as positive for CD. Using the manufacturer recommended cut-off values, sensitivity and specificity were 90–100% and 97–100% for EMA tests, whereas t-TG kits had 98% and 97%, respectively. Serum IgA levels were determined by nephelometric method, using commercially available kit (Siemens Healthcare Diagnostic Products Gmbh, Germany). IgA levels lower than 5 mg/dL were considered abnormal.

An upper digestive endoscopy with an intestinal biopsy (at least six biopsy samples obtained from the second duodenal portion) was proposed in case of antibodies positivity. CD diagnosis was made in presence of histological findings characterized by severe or partial villous atrophy along with crypt hyperplasia as indicated by Marsh [20]; histopathology was expressed according to Marsh criteria modified by Oberhuber et al. [21].

Moreover, each subject was asked to complete a questionnaire reporting the possible presence of one or more of the following conditions: infertility (defined as the failure to conceive after one year of regular intercourse without contraception), miscarriage, coexistence of other autoimmune diseases, and family history of autoimmune diseases.

### 2.1. Ethics Approval

Procedures were in accordance with the ethical standards of the Helsinki Declaration of 1964, as modified by the 48th World Medical Association General Assembly in 1996. Each subject gave written informed consent to the study. The study was approved by the Ethical Committee of the Catholic University of Rome (clinical trial registration number: Prot.cm. P588 (A.1138)/C.E./2008).

### 2.2. Statistical Analysis

Statistical comparison of patients and controls groups was performed by means of chi-square test or Fisher's exact test as appropriate. Statistical comparison of parametric variable (age) among the groups was performed by *t*-test for unpaired data. All values were expressed as total count and percentage. Age was expressed as mean ± standard deviation. A *P* value of 0.05 or less was regarded as significant.

## 3. Results

A total of 315 women with laparoscopic and histological confirmed diagnosis of endometriosis were considered during the whole period study. Ninety-two (29.2%) of them refused to participate in the study, and thus 223 (70.8%) patients were finally enrolled. According to the revised American Society for Reproductive Medicine classification of endometriosis, 30 (13.4%) patients were classified as having minimal endometriosis, 71 (31.8%) patients were classified as having mild endometriosis, 48 (21.5%) patients were classified as having moderate endometriosis, and 74 (33.2%) patients were classified as having severe endometriosis [19].

Two hundred and forty-six female nurses, without a known history of endometriosis, participated in the study as controls.

Mean age was 36 ± 6.6 years in the endometriosis group and 35.1 ± 7.7 years in the control group (*P* = 0.241).

Among all subjects with antibodies positivity, none refused the endoscopic examination.

No serum IgA deficiency was found in any subject (patients or controls).

According to serological and histological findings, CD occurrence was higher in patients with endometriosis than in controls, this difference being not statistically significant: 5 (2.2%) versus 2 (0.8%), respectively (*P* = 0.265) (Table 1). By dividing 5 subjects with both CD and endometriosis according to endometriosis classification [19], CD was found in 1 patient with minimal endometriosis, 2 patients with mild endometriosis, and 2 patients with moderate endometriosis.

Table 2 shows the distribution of medical records, as reported by patients and controls in the questionnaire: patients with endometriosis showed a largely higher rate of infertility compared to control group (27.4% versus 2.4%; *P* < 0.001); all the other considered conditions were similar between the two groups.

By dividing all study population according to presence/absence of CD, familiarity for autoimmune diseases and familiarity for CD were statistically more frequent among subjects with CD (71.4% versus 8.7%; *P* < 0.001, and 42.9 versus 1.9; *P* < 0.001, resp.).

## 4. Discussion

Our study reports a potential association between endometriosis and CD in Italian women, showing a trend for increased prevalence of CD in women affected by endometriosis, even if not statistically significant.

In the last few years, some recent studies about this topic have been performed, showing similar results [17, 18]. The interest arises from shared features of both CD and endometriosis, specifically concerning etiology and ongoing inflammation.

It is well known that CD is an autoimmune disorder in which an abnormal T cell response to gluten occurs. Dieterich et al. recently showed that the tissue enzyme transglutaminase is a target of immunological reaction, generating a complex of the prolamine of gluten with HLA molecule that is recognized by T helper cells [22]. It is noteworthy that CD is strongly associated with some HLA class II genes, in particular with homozygosis for HLA DQ2.5 haplotype; also a condition of heterozygosis for this haplotype associated with the presence of DQ2.2, DQ7, or DQ8 produces a higher risk of CD [23–25].

The presence of gut inflammation, resulting from the above-mentioned immunological response, together with abnormal intestinal permeability and consequent increased antigenic exposure and autoantibody production, could be also responsible for the association of CD with other autoimmune diseases: dermatitis herpetiformis, diabetes mellitus type 1, Sjögren's syndrome, rheumatoid arthritis, primary biliary cirrhosis, and sclerosing cholangitis. This theory is supported by the evidence that a number of autoantigens, normally cryptics, are processed and presented by APC to T cells, because of prolonged intestinal inflammation [26].

Another factor that could explain the association of CD with other autoimmune diseases is a common genetic background, represented by HLA haplotype; in fact, some genes in the region of major histocompatibility complex are involved in multiple autoimmune disorders, such as HLA DQA1 and DQB1 genes that can confer risk to both CD and type 1 diabetes [27].

Despite decades of extensive research, the pathogenesis of endometriosis remains not completely clarified. Actually, endometriosis is considered a multifactorial disorder, in which the* primum movens* seems to be represented by retrograde endometrial debris reflux into the peritoneal cavity that promotes increase of oxidative stress and consequent low-grade inflammation [3, 28, 29]. Peritoneal macrophages have been identified as key processes, by producing growth and angiogenic factors, as well as various proinflammatory cytokines that could be responsible for maintenance of disorder and impairment of reproductive function, as well as the systemic involvement that characterized endometriosis [30, 31]. In the last years, chronic inflammation with increased oxidative stress has been reported to be involved also in the association of endometriosis with other chronic inflammatory diseases, as atherosclerosis [32]. According to these evidences, endometriosis is now considered a chronic inflammatory disease, with inflammation not limited to peritoneal cavity but spread to systemic level, as signaled by elevated serum levels of markers as Ca-125 and C-reactive protein (CRP) [33, 34].

Also a genetic predisposition has been suggested for development and progression of endometriosis, with HLA DQ7 haplotype being reported as the first allele significantly associated with endometriosis [35, 36].

A potential role of autoimmunity for endometriosis has been suggested, as it fulfills many of the classification criteria of autoimmune diseases: polyclonal B cell activation, immunological abnormalities in T and B cell functions, defective apoptosis, tissue damage, and multiorgan involvement [37, 38]. This topic is supported also by familial occurrence, genetic predisposition, female preponderance, and increased likelihood of other autoimmune diseases, like systemic lupus erythematosus, rheumatoid arthritis, Sjogren's syndrome, multiple sclerosis, and endocrine disorders [8, 39].

Among these associations, recently some studies have evaluated the relationship with CD. The first study has determined the presence of CD in a Brazilian subpopulation of women with endometriosis suffering from infertility [17]. The authors have found that prevalence rates of positive CD serology for anti-tTG and antiendomysium IgA in a group of 120 patients with endometriosis versus 1500 controls were statistically significant (4.1% in patients versus 0.8% in controls), even if the prevalence rates of the biopsy-confirmed CD did not reach the statistical significance (2.5% cases versus 0.66% controls), showing only a positive trend, maybe also because one patient refused the endoscopic examination. They have concluded that, even if not statistically significant due to small number of cases, CD is more common in women with endometriosis with respect to controls, suggesting a potential clinical relevance. In the interpretation of their results, however, we cannot ignore that control group was constituted by blood donors; these subjects, as underlined by authors themselves, cannot be considered to be representative of the general population. In fact, some conditions, first of all the presence of anemia (one of the possible manifestations of CD), have to be excluded in subjects candidate as donors. Moreover, in this report total serum IgA assessment was not performed, making it not possible to exclude serum IgA deficiency, a condition that compromises the diagnostic power of serological assays for CD. Finally, since patients with endometriosis were enrolled among subjects referring for infertility disorders, women at higher risk of CD could have been screened, infertility being a complaint also of CD. As regards, in our study the enrollment was conducted among women with endometriosis, not necessarily involving the presence of infertility; however, an increased prevalence of infertility among patients with endometriosis with respect to controls was finally found.

Recently, a Swedish nationwide population-based study has evaluated the risk of developing endometriosis in about 11.000 women with known CD [18]. During the follow-up period of study, the authors have found an increased risk of developing endometriosis, hypothesizing that chronic inflammation characterizing CD could act as trigger in endometriosis development. It is not by chance that they reported that this risk was higher in the first year after the diagnosis of CD, when mucosal healing could not be yet achieved, despite gluten-free diet start. The greater awareness to CD presence, together with improved diagnosis and some socioeconomic factors, has led to increased prevalence of CD, especially identifying mild degrees of CD; pursuant to authors' view, pointing severity of inflammation due to CD to be crucial for endometriosis development, the presence of mild CD could modify the association with endometriosis. As regards, in our study all subjects diagnosed as affected by CD (both patients and controls), although clinically not suspected for CD, presented villous atrophy with high grades of inflammation at intestinal biopsy.

In conclusion, our results confirm the potential association between CD and endometriosis, although this trend does not reach the statistical significance. Further studies with higher number of subjects are desirable to definitively support this relationship. Actually, we can suggest screening a woman with endometriosis for CD if a valid clinical suspect is present.

## Figures and Tables

**Table 1:** Serological and histological findings in patients and controls.

	Endometriosis N° 223	Controls N° 246	*P*
Only EMA Ab positivity	0 (0%)	0 (0%)	1
Only t-TGA Ab positivity	0 (0%)	0 (0%)	1
Both EMA and t-TGA Ab positivity	5 (2.2%)	2 (0.8%)	0.265
Marsh-Oberhuber classification			
3a	2 (0.9%)	2 (0.8%)	1
3b	3 (1.3%)	0 (0%)	0.11

**Table 2:** Distribution of medical records as reported in the questionnaire in patients and controls.

Condition	Endometriosis N° 223	Controls N° 246	*P*
Infertility	61	6	<0.001
	(27.4%)	(2.4%)	
Abortivity	30	28	0.906
	(13.5%)	(11.4%)	
Autoimmune disease	20	17	0.838
	(9.0%)	(6.9%)	
Familial history of autoimmune disease	34	35	0.567
	(15.2%)	(14.2%)	
Familial history of celiac disease	12	11	0.978
	(5.4%)	(4.5%)	
